# The resilience scale: factorial structure, reliability, validity, and parenting-related factors among disaster-exposed adolescents

**DOI:** 10.1186/s12888-021-03153-x

**Published:** 2021-03-10

**Authors:** Xuliang Shi, Shuo Wang, Zhen Wang, Fang Fan

**Affiliations:** 1 School of Education, Hebei University, Baoding, 071002 Hebei China; 2 School of Public Administration, Xinhua College of Sun Yat-sen University, Dongguan, 510520 Guangdong China; 3 School of Psychology, Center for Studies of Psychological Application, and Key Laboratory of Mental Health and Cognitive Science of Guangdong Province, South China Normal University, Guangzhou, 510631 China

**Keywords:** Psychometric properties, Factors, Resilience scale, Adolescents, Earthquake

## Abstract

**Background:**

In this study, we examined psychometric properties of the Chinese version of the Resilience Scale (RS) and parenting-related factors associated with resilience among disaster-exposed adolescents.

**Methods:**

Eighteen months after the earthquake, a total of 1266 adolescents (43.4% male, mean age = 15.98; SD = 1.28) were assessed using the RS, the Post-traumatic Stress Disorder Self-Rating Scale, the Depression Self-rating Scale for Children, the Screen for Child Anxiety Related Emotional Disorders, and Parental Bonding Instrument.

**Results:**

Through exploratory factor analyses (EFAs) and parallel analysis, responses were characterized into a 3-factor structure: personal competence, meaningfulness, and acceptance of self and life. Cronbach’s alpha coefficient for the RS was 0.89 and the test-retest reliability coefficient was 0.72. In terms of predictive validity, resilience was found to be a significant predictor for PTSD, depression, and anxiety. Multiple regression analysis showed that maternal parenting styles were significant predictors of resilience after adjusting for gender, age, sibling number, and earthquake experiences.

**Conclusions:**

The Chinese version of RS is a reliable and valid tool for assessing resilience among adolescent survivors after disasters. The implications for research and resilience-oriented interventions were also discussed.

## Background

Adverse experiences (e.g., natural disasters, war, terrorism, and sexual/physical abuse) during childhood or adolescence can result in long-term impairment of one’s psychosocial functioning [1–4]. Although most people encounter various adverse or traumatic events in the course of their lives, their reactions to such events are not universal [5]. Some people may exhibit psychological difficulties, while others actively respond to disasters, and may even attain post-traumatic growth. Resilience is defined as the ability to bounce back from social disadvantages or highly adverse conditions. Elucidating the role of resilience in children and adolescents in the aftermath of traumatic events can help improve their well-being and suppress the risk of developing psychopathologies, including posttraumatic stress disorder (PTSD), depression, and anxiety [6–8].

Despite the fact that there is no universally accepted definition of resilience, two core concepts have been previously mentioned: adversity and positive adaptation. Bonanno defined resilience as the ability to maintain a relatively stable psychological and physical functioning during adversity [5]. Based on increasing interest in this concept, many scales have been developed to assess resilience in development psychology, including the Resilience Scale (RS) [9], Connor-Davidson Resilience Scale (CD-RISC) [10], Resilience Scale for Adults (RSA) [11], and Brief Resilience Coping Scale (BRCS) [12]. Among them, one scale widely used in prior studies is the Resilience Scale [13].

The Resilience Scale (RS) was initially developed from a qualitative study [9]. In this study, 24 elderly women who had demonstrated a successful adaptation to traumatic events were interviewed. Five components (equanimity, perseverance, meaningfulness, self-reliance, and existential aloneness) were found to be correlated with resilience. In an empirical study [9], two factors (personal competence and acceptance of self and life) were extracted from 25 RS items through exploratory factor analysis. Personal competence consists of 17 items including self-reliance, perseverance, mastery, and resourcefulness, while acceptance of self and life includes 8 items involving acceptance of life and a sense of peace in spite of adversities. The 25-item RS has been translated into many languages, including Chinese, Dutch, Swedish, and Japanese, all of which have shown good psychometric characteristics [7, 14–16]. Studies have documented that the mental structure of resilience may vary depending on cultural contexts. For example, 5 factors were identified in the Swedish version [15], whereas 3 factors were extracted from the Brazilian version [17]. Furthermore, inconsistent results from different samples of the same country have been documented. For instance, after the 2010 Haitian earthquake, a 5-factor model was found to be supported by child and adolescent survivors [18], whereas a 3-factor model was confirmed by adult survivors [19]. Therefore, it is important to examine the psychometric properties of the 25-item RS under different socio-cultural backgrounds.

Risk and protective factors associated with resilience are of particular importance. According to the current resilience models, the factors affecting resilience can be grouped into internal and external factors. Internal factors, such as demographic (gender and age) and psychological (optimism and gratitude) factors, are generated from within an individual. External factors, such as family functions and school climate, are generated from outside of a person. Among the external factors, parenting styles are of particular importance. Parenting styles can affect an adolescent’s mental health [20, 21]. Positive parenting styles, such as warm, supportive, and acceptance, have been associated with increasing resilience, for example, in protecting adolescents from depression and anxiety. In a study involving elementary school children, it was found that an authoritative parenting style was negatively associated with parent- and teacher-rated maladaptive behavior, and positively correlated with indicators of healthy adjustment [22]. In the Chinese culture, mothers spend more time taking care of their children compared to fathers, and children tend to feel closer to their mothers than their fathers. Mothers are more involved in educating their children, and thus, maternal parenting styles may exert a greater impact on children’s adjustment. The above studies are limited by the fact that they mainly involved normal children and adolescents. These associations have not been elucidated in traumatized adolescents. Based on this limitation, we systematically examined the contribution of maternal parenting styles to resilience among adolescent survivors after a natural disaster, which may inform the development of interventions aimed at improving resilience.

At 14:28 on the 12th of May, 2008, a destructive earthquake occurred in Sichuan province of southwest China. It resulted in 69,227 deaths, 374,176 injuries and 18,222 missing people. A great number of the children and adolescents suffered the direct effects of the earthquake. Six months after the earthquake, a cohort study was conducted in Dujiangyan city, one of the most severely damaged areas [23]. With the data on resilience and parenting styles among adolescent survivors from this cohort, the objectives of the current study were: i. To examine the underlying structure and the psychometric properties of the 25-item RS in the Chinese context; and ii. To assess the resilience levels and determine whether maternal parenting styles are important predictors of resilience among adolescent survivors.

## Materials and methods

### Participants and study design

This study was part of the Wenchuan Earthquake Adolescent Health Cohort (WEAHC). In WEAHC, participants were surveyed for a total of five times at an interval of 6 months. All surveys were conducted between December, 2008 and January, 2011. Sampling and data collection methods were as previously described [23]. Briefly, a total of 1573 adolescents affected by the Wenchuan earthquake were surveyed at baseline (about 6 months after the earthquake). In this study, data from the third survey conducted in December 2009 (about 18 months after the earthquake) was used. A total of 1266 students were followed up at this stage. Among the students who participated, 43.4% were males, with a mean age of 15.98 ± 1.28 years. Most of the students (83.3%) were the only child in their families. With respect to earthquake exposure, 25.0% reported dead, missing, or injured family members; 58.8% witnessed traumatic scenes; 42.6% reported severe house damage; while 21.3% suffered the loss of family property.

Self-administered questionnaires were administered to target students in classroom settings. The students were informed that they were free to withdraw from the study at any time. The questionnaires were filled at an average of 30 min per student. The study methods were performed in accordance with the relevant guidelines and regulations. Ethical approval for this study was obtained from the Human Research Ethics Committee of South China Normal University, with permission and support from the participating school boards and the Chengdu Women’s Federation. A written informed consent was also obtained from the participating students and their parents, or guardians.

### Measures

#### Demographic characteristics

The collected demographic information included gender (0 = male, 1 = female), age, and sibling number (0 = more than one child, 1 = only one child).

#### Earthquake experiences

Four items were used to evaluate the participants’ earthquake experiences at baseline: i. Dead, missing, and/or injured family members; ii. House damage; iii. Property loss (other than house damage); and iv. Direct witness of the tragic disaster. The first three items were scored on a 5-point scale ranging from 1 (the highest level of exposure) to 5 (the lowest level of exposure). The last item was scored as: 1 = not directly seeing the disaster scene and 2 = directly seeing the disaster scene. Item scores were added to create a composite score for earthquake exposure, with higher scores (ranging from 4 to 17) indicating higher exposure levels.

#### Resilience

The Chinese version of Resilience Scale was used to assess resilience levels [9, 24]. With permission from the original authors, the scale was translated into Chinese and then, back-translated by one English teacher and two graduate students majoring in psychology [24], until a consensus was reached. This scale includes 25 items and participants were asked to respond based on a 7-point Likert scale ranging from strongly disagree (scored 1) to strongly agree (scored 7), with a higher score representing a higher resilience level. The Chinese version of RS has been shown to have adequate psychometric properties for Chinese adolescents [24]. The internal consistency of this RS version was 0.89, while the test-retest reliability after two weeks was 0.74. In this study, Cronbach’s alpha was 0.93.

#### Posttraumatic stress disorder symptoms

The Post-traumatic Stress Disorder Self-Rating Scale (PTSD-SS) was used to evaluate PTSD symptoms [25]. The PTSD-SS was developed in accordance with the diagnostic criteria of PTSD as described in DSM-IV and the Chinese Classification of Mental Disorders. This scale entails 24 items, each rated on a 5-point scale varying from 1 (“not at all”) to 5 (“extremely severe”). The item scores were added to generate a total score ranging from 24 to 120. The PTSD-SS exhibits satisfactory psychometric properties for Chinese adolescents [25]. In the current study, Cronbach’s alpha was 0.95.

#### Depression symptoms

Depression symptoms were measured using the Depression Self-rating Scale for Children (DSRSC), which has good reliability and validity for use in Chinese children and adolescents [26, 27]. This scale consists of 18 items, each rated on a 3-point scale (0 = never, 1 = sometimes, 2 = mostly). A higher total score (ranging from 0 to 36) indicates a higher level of depression. In the current study, Cronbach’s alpha was 0.82.

#### Anxiety symptoms

The Chinese version of the Screen for Child Anxiety Related Emotional Disorders (SCARED) is a 41-item self-report measure designed to screen for anxiety disorders among children and adolescents [28, 29]. The items were rated on a 3-point scale (0 = almost never, 1 = sometimes, 2 = often). A total score ranging from 0 to 82 was used in this study. In the current study, Cronbach’s alpha was 0.94.

#### Maternal parenting styles

Parental Bonding Instrument (PBI) was used to assess adolescents’ perception of parenting styles. In this sample, most of the children’s fathers were absent due to work related factors. Thus, we only examined the influence of maternal parenting styles on resilience. This scale includes three subscales: i. Care (e.g. Spoke to me in a warm and friendly voice); ii. Autonomy (e.g. Let me do those things I liked doing)”, and ii. Overprotect (e.g. Tried to control everything I did)” [30, 31]. The first two subscales reflect positive parenting styles, while the last one reflects negative parenting styles. Care refers to the mother’s affection and emotional warmth, empathy, and closeness. Autonomy reflects that mothers encourage their children to be independent. Overprotection reflects mother’s excessive interference and control in children’s behavior. This scale consists of 23 items (care: 11 items; autonomy: 6 items; overprotect: 6 items) rated on a 4-point scale from 0 (“very unlike”) to 3 (“very like”). The total score of the three subscales was used in the subsequent analysis. In the current study, Cronbach’s alpha was 0.69.

### Analysis design

Descriptive statistics were used to describe the total scores of resilience, while *t*-tests were separately performed to test the differences in gender and siblings on the total resilience score. To facilitate exploratory and confirmatory factor analysis (EFA & CFA), the participants were assigned into two subsamples using a split-sampling random procedure in SPSS. Subsample 1 (*N* = 628) was used for EFA while subsample 2 (*N* = 638) was used for CFA. In EFA analysis, principal component analysis (PCA) with oblique rotation was performed to obtain factor solution. Scree plot and parallel analysis Monte Carlo PCA for parallel analysis [32]; was used to determine the number of factors to be extracted. Items with factor loading greater than 0.40 were retained. In CFA analysis, these indices were used to assess the goodness of model fit: the comparative fit index (CFI), the Tucker-Lewis index (TLI), the root mean square error of approximation (RMSEA), and the standardized root mean residual (SRMR). Cronbach’s α coefficients were used to determine internal consistency, and the test-retest reliability was evaluated using data from 200 participants, 6 months later. To assess the predictive validity of RS, a series of regression analyses were performed with the three mental disorders (PTSD, depression, and anxiety) as dependent variables. Third, multiple regression was performed to determine whether maternal parenting styles are important resilience predictors. All statistical analyses were performed using SPSS 23.0 and Mplus 7.4. All reported *p* values were two-sided, and *p ≤* 0.05 was considered to be statistically significant.

## Results

### Descriptive statistics

The average resilience score was 110.89 ± 26.25. The *t*-test for independent samples revealed that male adolescents reported higher levels of resilience than females (*t* = 3.18, *p* <  0.01, Cohen’s d = 0.18). Participants who had no siblings exhibited greater resilience than those with siblings (*t* = 2.36, *p* <  0.05, Cohen’s d = 0.19). In addition, the item-total correlation coefficient of the 25 items were all above 0.40, and ranged from 0.42 to 0.69 (see Table 1).

**Table 1:** Resilience Scale: item means, standard deviations, item-total correlation and factor loadings

Items ^a^	*M*	*SD*	Item-total correlation ^b^	Factor 1^c^	Factor 2	Factor 3
I am determined. (10)	4.08	1.79	0.57	0.63		
I seldom wonder what the point of it all is. (11)	4.74	1.62	0.63	0.63		
I can get through difficult times because I have experienced difficulty before. (13)	4.80	1.67	0.65	0.60		
I can be on my own if I have to. (5)	5.56	1.57	0.51	0.60		
I usually take things in stride. (7)	4.62	1.67	0.69	0.59		
I take things one day at a time. (12)	4.13	1.69	0.63	0.58		
I feel that I can handle many things at a time. (9)	4.04	1.68	0.57	0.56		
I am able to depend on myself more than anyone else. (3)	4.60	1.70	0.43	0.53		
I have self-discipline. (14)	4.57	1.78	0.57	0.47		
I feel proud that I have accomplished things in life. (6)	5.50	1.55	0.56	0.45		
When I’m in a difficult situation, I can usually find my way out of it. (23)	4.51	1.54	0.65	0.44		
When I make plans, I follow through with them. (1)	3.74	1.63	0.50	0.43		
I usually manage one way or another. (2)	4.69	1.65	0.55	0.42		
I have enough energy to do what I have to do. (24)	4.74	1.70	0.65	0.42		
I keep interested in things. (15)	4.73	1.78	0.57		0.80	
I can usually find something to laugh about. (16)	5.21	1.75	0.56		0.72	
Keeping interested in things is important to me. (4)	4.73	1.89	0.53		0.69	
My life has meaning. (21)	5.01	1.76	0.57		0.58	
I am friends with myself. (8)	4.96	1.82	0.58		0.53	
My belief in myself gets me through hard times. (17)	4.79	1.70	0.61		0.52	
Sometimes I make myself do things whether I want to or not. (20)	3.98	1.83	0.42			0.61
I do not dwell on things that I can’t do anything about. (22)	4.35	1.99	0.43			0.52
It’s okay if there are people who don’t like me. (25)	4.48	2.07	0.42			0.51
I can usually look at a situation in a number of ways. (19)	4.52	1.61	0.59			0.50
In an emergency, I’m someone people can generally rely on. (18)	4.23	1.67	0.51			0.43
Eigenvalue				7.83	1.48	1.37
Variance explained (%)				31.30	5.93	5.49

Note: ^a^ All items of Resilience Scale were presented. ^b^ All item coefficients were significant at the alpha 0.01 level. ^c^ Coefficients below 0.40 are not displayed in the table

### Exploratory and confirmation factor analysis of the Chinese version of RS

**Fig. 1:**
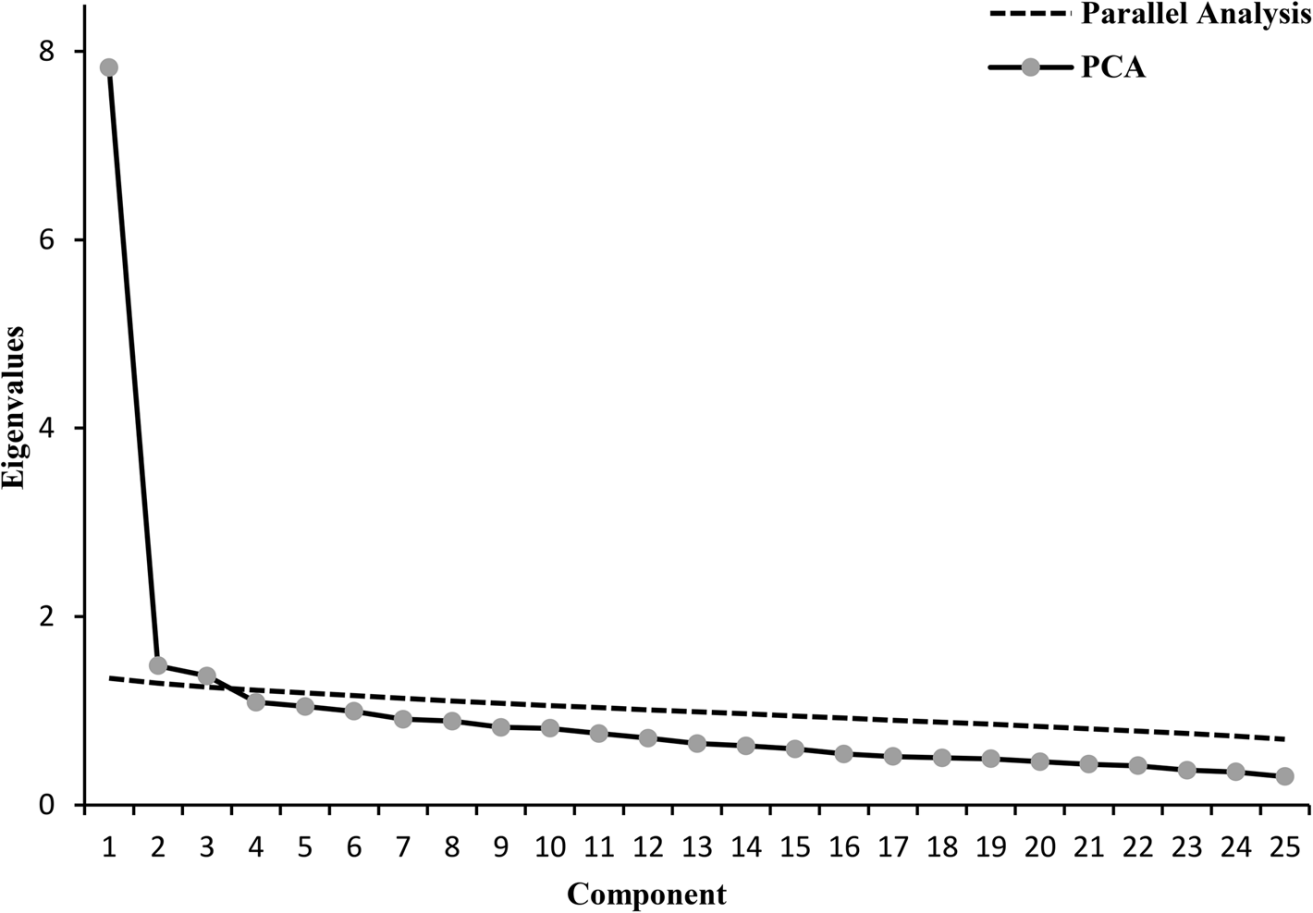
Scree plot and parallel analysis of eigenvalues based on principal component analysis of the Resilience Scale

Exploratory factor analysis was performed to explore the factor structure for RS. The Kaiser-Meyer-Olkin (KMO) value was 0.83 while the Bartelett sphericity test was significant (*χ*^2^ = 3671.39, *df* = 300, *p* <  0.001), suggesting that the data was suitable for EFA. Five factors had an eigenvalue higher than 1. However, the scree plot and parallel analysis allowed us to retain a three-factor model (Fig. 1), which accounted for 42.72% of the total variance. The factor loadings are shown in Table 1.

Factor 1 was labeled “personal competence”, composed of 14 items reflecting self-reliance, mastery, and perseverance when people are faced with trauma. Factor 2 was labeled “meaningfulness”, consisting of 6 items reflecting an individual’s sense for having something for which to live. Factor 3 with 5 items was labeled “acceptance of self and life”, suggesting flexibility, adaptability and a sense of peace in spite of adversity.

CFA for the 3-factor models in Sample 2 was performed to confirm the factor structure of the RS among Chinese adolescent survivors. The model was significant, *χ*^2^ (272) = 1090.75, *p* <  0.001, which could be attributed to the large sample size. In addition, RMSEA = 0.04, CFI = 0.95, TLI = 0.96, and SRMR = 0.04, suggested an acceptable fit model. Moreover, we examined the two-factor model derived from Wagnild and Young [9] and the five-factor model with an eigenvalue higher than 1. EFA analyses revealed a poor fit for these two models (fit indices of two-factor model: *χ*^2^ (274) = 1524.68, *p* <  0.001, CFI = 0.85, GFI = 0.87, and RMSEA = 0.08; fit indices of five-factor model: *χ*^2^ (265) = 1275.48, *p* <  0.001, CFI = 0.81, GFI = 0.74, and RMSEA = 0.15).

### Internal consistency and test-retest reliability of the Chinese version of RS

The internal consistency of the total RS was 0.89, while Cronbach’s alpha coefficients of the 3 factors were 0.89, 0.83, and 0.79, respectively. These findings imply a relatively good internal consistency. The correlations among the three factors were significant at 0.01, ranging from 0.44 to 0.64. For the test-retest reliability, a total of 200 adolescents from the third survey were reevaluated in June, 2010 (about 24 months after the earthquake). The test-retest reliability coefficient (6-month interval) was found to be 0.72 for the whole scale, and 0.70, 0.75 and 0.68 for the 3 factors, respectively.

### Predictive validity

To assess the predictive validity of RS, a series of regression equations were run. In the regression analysis, mental disorders (including PTSD, depression, and anxiety) were taken as dependent variables and resilience as independent variable. As shown in Table 2, after adjusting for gender, age, sibling number, and earthquake experiences, resilience significantly predicted PTSD (*β* = − 0.16, *p* <  0.001), depression (*β* = − 0.35, *p* <  0.001), and anxiety symptom (*β* = − 0.25, *p* <  0.001).

**Table 2:** Regression analysis evaluating the predictive validity of RS on mental disorders

Outcome variables ^a^	B	S.E.	*β*	*t*	*p*
PTSD	− 0.09	0.02	− 0.16	− 5.77	< 0.001
Depression	− 0.09	0.01	−0.35	−13.47	< 0.001
Anxiety	−0.15	0.02	−0.25	−9.40	< 0.001

Note: RS = Resilience Scale. ^a^ Adjusted for gender, age, sibling number and earthquake experiences

### Maternal parenting styles as predictors of resilience

As shown in Table 3, after adjusting for gender, age, sibling number, and earthquake experiences, participants who reported perceiving more maternal care (*β* = 0.22, *p* < 0.001), more mothers’ autonomy-relevant parenting (*β* = 0.22, *p* < 0.001), and less maternal overprotect (*β* = − 0.16, *p* < 0.001) had higher levels of resilience.

**Table 3:** Multiple regression analysis predicting resilience

Predictors ^a^	B	S.E.	*β*	*t*	*p*
Mother-care	1.61	0.23	0.22	7.13	< 0.001
Mother-autonomy	1.39	0.19	0.22	7.35	< 0.001
Mother-overprotect	−1.25	0.24	−0.16	−5.15	< 0.001

Note: ^a^ adjusting for gender, age, sibling number, and earthquake experiences

## Discussion

The first objective of this study was to evaluate the psychometric properties of the Chinese version of the RS in 1266 adolescent survivors of the Wenchuan earthquake. Factor analysis showed that a 3-factor model (factor 1: personal competence; factor 2: meaningfulness; factor 3: acceptance of self and life) was acceptable. Personal competence refers to the willingness or belief that individuals should rely on their own strengths to reconstruct their lives when they encounter setbacks. Meaningfulness refers to the realization that life has a purpose and the sense of having something for which to live. Acceptance of self and life means that individuals can always appreciate life and a sense of peace in the face of adversity. Compared to the study by Wagnild and Young [9], there is a similarity between their factors and our two factors (factor 1 and factor 3). These two factors were also validated using a sample of adult survivors following Haiti’s earthquake [19]. In addition, the second factor (meaningfulness) in our study was also presented within the five characteristics of resilience as defined by the original authors [9]. This factor measured the extent by which the belief in having a goal in life is established. It is the recognition that there is something for which to live.

The reliability coefficient of the RS in our study was 0.89, which was comparable to other reliability studies [7, 14]. As noted in a previous review, Wagnild [33] also found that the internal consistency of the RS was consistently high in 11 of 12 reviewed studies (Cronbach’s alpha coefficient ranged from 0.85 to 0.94). In addition, the test-retest coefficient of our study was 0.72, which was lower than the results of previous studies. For example, the test-retest correlation (1-month interval) was 0.78 in the study by Nygren, et al. [34], and 0.82 (6-month interval) in the study by Lei, et al. [7] . Since a limited number of studies have evaluated the test-retest reliability of the RS, more studies should be performed to test the stability of RS. Regarding the predictive validity of RS, we found that resilience predicted PTSD, depression, and anxiety after adjusting for some confounders. Compared to individuals with low resilience, individuals with a high resilience exhibited fewer PTSD, depression, and anxiety symptoms. These results are consistent with those of previous studies [35, 36]. For example, three months after the Jiuzhaigou earthquake, a total of 607 participants were recruited from the heavy damaged areas and assessed. It was found that resilience was significantly correlated to the severity of PTSD, depression, and anxiety symptoms [37]. These correlations were attributed to several factors. First, highly resilient adolescents often adopt effective cognitive emotion regulation strategies [38] that serve to attenuate the impact of mental illness. Second, resilient adolescents have positive emotions [39] and optimistic attitudes [40], all of which would contribute to reducing the impact of mental illness.

After the earthquake, male adolescents showed higher levels of resilience than females, inconsistent with previous studies that did not find gender-related differences [18, 33]. However, our finding was in agreement with that of Lei, et al. [7] who reported that male college students had higher resilience scores than females. Moreover, previous studies that used the Connor-Davidson Resilience Scale also found that male participants had significantly higher resilience scores than females [41, 42]. This could be attributed to the fact that, compared to males, females have abnormal HPA responses to stress, which may makes them more sensitive to stress [43]. The participants who had no siblings reported greater resilience than those with siblings. Inferably, compared to children with siblings, the only child might receive extensive attention and support from family members or other closed ones.

The second objective of this study was to determine whether maternal parenting styles had unique contributions to resilience. Teenagers whose mothers preferred caring and autonomy-relevant parenting had higher levels of resilience. This kind of mother considers the children’s wish and opinions when appropriate, gives them a positive feedback and corrects their negative behaviors. These results are consistent with those of previous studies [44, 45]. For example, one South African study involving a sample of 360 teenagers found that authoritative parenting, characterized by warmth and democracy, was one of the most important predictors of resilience [44]. Similar results were also reported in elderly adults [45]. Moreover, overprotected teenagers were found to have a lower resilience. This result was consistent with previous studies [46, 47]. For instance, in a sample of 200 college students and their parents, the researchers found that perceptions of maternal control were a powerful predictor for students’ anxiety [48]. Overprotective mothers always try to protect their children against experiencing life or enduring struggles that make them anxious, which might hamper their independence and self-confidence, thus reducing their self-efficacy and resilience.

Several limitations should be noted when interpreting the findings. First, the measurements of resilience in this study were self-reported by adolescents, which may be biased by social desirability. Multiple assessments (e.g. parents’ reports or teachers’ reports) should be considered in future studies. Second, participants in this study were only recruited from among adolescents who had suffered a devastating earthquake, which limits generalization of the conclusions to adult populations and other types of acute or chronic traumatic events. It is also possible that the sampling range may bias the validity of the factors. These findings should be verified using larger and representative samples. Third, resilience is a multidimensional concept that may be affected by a variety of factors, such as biological, psychosocial, or contextual. Future studies should use multiple markers that can increase or decrease resilience.

Notwithstanding these limitations, this study shows that the RS is a reliable and valid tool for assessing resilience among adolescent survivors after disasters. Resilience scores may be useful for identifying vulnerable individuals at a high risk of developing mental disorders following natural disasters, so that they might be referred for mental health services. The scale can be administered to a large number of individuals at a low cost, and can maximize the utilization of limited resources in the occurrence of natural disasters. Large scale screening methods may not only be more cost-effective, but can also reduce stigma and discrimination, which might be induced by using scales of psychiatric symptoms as criteria for referral to services. This study also contributes to the identification of factors associated with resilience, which may provide some insights for the development of resilience-oriented interventions. Interventions aimed at enhancing resilience can be carried out in school settings. These interventions have been shown to be effective in reducing the symptoms of depression and anxiety [49]. Therefore, from a public health perspective, resilience-oriented prevention and early intervention programs are a relatively low-cost investment with the potential for excellent returns.

## Conclusions

The Chinese version of the Resilience Scale (RS) has good psychometric properties, and the 3-factor model (factor 1: personal competence; factor 2: meaningfulness; factor 3: acceptance of self and life) fits best. In addition, multiple regression analysis showed that maternal parenting styles are significant predictors for adolescent survivors’ resilience.

## Data Availability

The datasets used and/or analyzed during the current study are available from the corresponding author on reasonable request.
